# Changes in Tree Reproductive Traits Reduce Functional Diversity in a Fragmented Atlantic Forest Landscape

**DOI:** 10.1371/journal.pone.0000908

**Published:** 2007-09-19

**Authors:** Luciana Coe Girão, Ariadna Valentina Lopes, Marcelo Tabarelli, Emilio M. Bruna

**Affiliations:** 1 Programa de Pós-Graduação em Biologia Vegetal, Departamento de Botânica, Universidade Federal de Pernambuco, Recife, Pernambuco, Brazil; 2 Departamento de Botânica, Universidade Federal de Pernambuco, Recife, Pernambuco, Brazil; 3 Department of Wildlife Ecology and Conservation, University of Florida, Gainesville, Florida, United States of America; Centre National de la Recherche Scientifique, France

## Abstract

Functional diversity has been postulated to be critical for the maintenance of ecosystem functioning, but the way it can be disrupted by human-related disturbances remains poorly investigated. Here we test the hypothesis that habitat fragmentation changes the relative contribution of tree species within categories of reproductive traits (frequency of traits) and reduces the functional diversity of tree assemblages. The study was carried out in an old and severely fragmented landscape of the Brazilian Atlantic forest. We used published information and field observations to obtain the frequency of tree species and individuals within 50 categories of reproductive traits (distributed in four major classes: pollination systems, floral biology, sexual systems, and reproductive systems) in 10 fragments and 10 tracts of forest interior (control plots). As hypothesized, populations in fragments and control plots differed substantially in the representation of the four major classes of reproductive traits (more than 50% of the categories investigated). The most conspicuous differences were the lack of three pollination systems in fragments-pollination by birds, flies and non-flying mammals-and that fragments had a higher frequency of both species and individuals pollinated by generalist vectors. Hermaphroditic species predominate in both habitats, although their relative abundances were higher in fragments. On the contrary, self-incompatible species were underrepresented in fragments. Moreover, fragments showed lower functional diversity (H' scores) for pollination systems (−30.3%), floral types (−23.6%), and floral sizes (−20.8%) in comparison to control plots. In contrast to the overwhelming effect of fragmentation, patch and landscape metrics such as patch size and forest cover played a minor role on the frequency of traits. Our results suggest that habitat fragmentation promotes a marked shift in the relative abundance of tree reproductive traits and greatly reduces the functional diversity of tree assemblages in fragmented landscapes.

## Introduction

Functional diversity can be defined as a variety of life-history traits presented by an assemblage of organisms [1], [2] and it has been postulated to be critical for the maintenance of ecosystem processes and properties [3]. For example, previous empirical work has suggested that ecosystems with a high diversity of functional traits have greater efficiency of water, nutrient, and light use, as well as higher productivity [3], [4]. In addition, they may also be more resilient [5] and resistant to biological invasions and to biodiversity loss [6], [7]. Nevertheless, most studies on functional diversity in plant communities have focused on the importance of traits associated with plant physiology. Consequently, we know little regarding the functional diversity of other traits that also affect both community structure and ecosystem functioning, such as those related to plant-animal interactions [1], [2].

Habitat loss and fragmentation (hereafter habitat fragmentation) have been shown to dramatically alter tree communities in tropical forests [8]–[12]. Fragments usually exhibit reduced species richness and diversity, particularly near edges. This reduction in species diversity is due in large part to loss of species that are “shade-tolerant” [8], [12], [13], restricted to the forest understory [10], have large-seeds [14], [15], or are dispersed by vertebrates [12], [16]–[19]. Furthermore, fragments tend to become dominated, both in terms of species richness and individual abundance, by pioneer trees [8], [19]. Because tropical pioneer trees usually share a similar set of life-history traits irrespective of their taxonomic affinities [20]–[22], this biased ratio of pioneers to shade-tolerant plants may reduce the functional diversity of tree assemblages in fragments.

More than 90% of the extant angiosperms are animal-pollinated [23], therefore pollination is considered an essential ecosystem process whose outcome can have major consequences for the maintenance of biodiversity [24], [25]. Indeed, a broad body of empirical evidence has found that the disruption of plant-pollinator interactions by habitat fragmentation can detrimentally affect plant reproductive success [26]–[29]. Potentially, changes in plant-pollinator interactions and pollinator abundance/composition can affect seed dispersal and seedling recruitment and consequently reduce plant population size or even promote local extinction [26], [27], [30]. Nevertheless, patterns and process regarding changes in reproductive functional diversity in fragmented tropical landscapes remain poorly investigated.

Because the long lifespan of tropical trees [31], hypotheses addressing disruptions of functional diversity driven by changes in tree composition can be properly tested in landscapes that were disturbed long enough ago to permit demographic shifts to have occurred, such as fragmented landscapes with longer histories of human occupation. The Atlantic forest of Brazil is a biodiversity hotspot that has been reduced to less than 8% of its original distribution due to forest clearing and fragmentation that dates to the 16^th^ century [32]. In some regions (*e.g.* Brazil's northeast), over 90% of fragments are smaller than 50 ha and are immersed in a homogeneous and hostile matrix of sugar cane fields [33]. These archipelagos of small fragments and forest edge habitat are currently dominated by a small subset of pioneer trees, retain less than half of the tree species richness of the forest interior [19], and receive an impoverished seed rain biased towards smaller seeds [34]. This scenario offers an excellent opportunity to investigate long-term fragmentation-related changes in tree assemblages and how they influence functional diversity.

Here we test the hypothesis that the habitat fragmentation changes the frequency of tree species and individuals within categories of reproductive traits and consequently reduces the functional diversity of tree assemblages in a fragmented landscape of the Brazilian Atlantic forest. We begin by comparing the pollination systems, floral biology, sexual systems, and reproductive systems of trees in forest fragments and tracts of forest interior (control plots). We then compare the diversity of these traits in these two habitats based on the relative contribution of both species and individuals. Finally, we discuss potential mechanisms driving the patterns we observed. We conclude that habitat fragmentation promotes a marked shift in the relative abundance of tree reproductive traits, including the lack of some specialized pollination systems and a parallel increase in the frequency of generalist ones. Collectively, shifts in reproductive traits promote a conspicuous reduction in the functional diversity of tree assemblages in fragmented landscapes, which may strongly influence forest dynamics and the persistence of biodiversity.

## Materials and Methods

### Study site and landscape attributes

**Figure 1:**
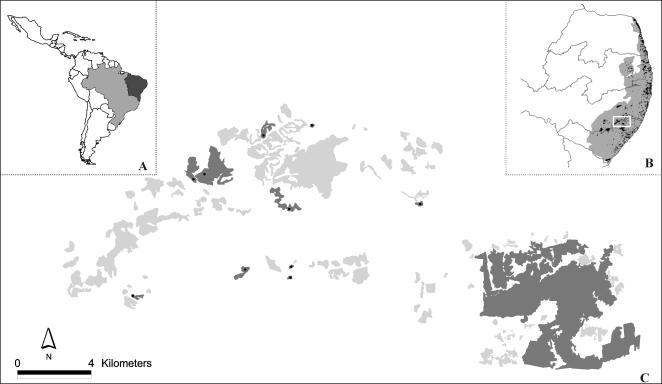
Study landscape at the Atlantic forest of northeast Brazil. (A) Northeastern Brazil, where this study was conducted. (B) Distribution of the Atlantic forest of northeast Brazil ( = Pernambuco Center of Endemism), note original (grey) and current (black) distribution of this forest in the region; white rectangle represents the study landscape (amplified in C). (C) Study landscape with the fragments used in this study (dark grey polygons), including the 3,500 ha Coimbra forest (lower right). Light grey and white areas represent remaining forest fragments (not sampled) and sugar-cane cultivation, respectively.

This study was conducted in the State of Alagoas in northeastern Brazil on the property of Usina Serra Grande (8°58′50″S, 36°04′30″W), a large, privately-owned sugar producer. This landholding has approximately 9,000 ha of forest included in a unique biogeographic region of the Atlantic forest known as the Pernambuco Center of Endemism [*sensu* 35] or the Atlantic forest of Northeast Brazil, the most threatened sector of the South American Atlantic forest [17]. We selected a large (666.7 km^2^), severely fragmented landscape within this property containing 109 forest fragments (total forest cover = 9.2%), including the 3,500-ha Coimbra forest–the largest and best preserved remnant in this region [19]. All fragments are entirely surrounded by a uniform matrix of sugar-cane monoculture (Figure 1). In addition to the Coimbra forest, the patches ranged in size from 1.67–295.7 ha.

Our study landscape consists of a low-altitude plateau (300–400 m above sea level) containing two similar classes of dystrophic soils with high clay fractions: yellow-red latosols and yellow-red podzols (according to the Brazilian soil classification system [36]). Annual rainfall is ∼2000 mm, with a 3-month dry season (<60 mm/month) from November to January. Forests in this landscape consist of lowland *terra firme* forest (<400 m a.s.l.) [37], with the Fabaceae, Lauraceae, Sapotaceae, Chrysobalanaceae and Lecythidaceae accounting for most tree species (≥10 cm DBH) [38], [39]. Sugar cane cultivation in this landscape, which dates to the early 19^th^ century, and possibly as early as the 18^th^ century [see 40], provided the strongest incentive for clearing large tracts of pristine old-growth forests. Remaining forest fragments have been protected against fire and logging to ensure watershed protection and water supply for sugar cane irrigation (C. Bakker, pers. communication). This protection has guaranteed the stability of forest fragment borders and the occurrence of both pioneer and shade-tolerant adult trees along forest edges as evidenced by local patterns of seed rain [15]. The Serra Grande landscape therefore provides a rare and interesting opportunity for Atlantic forest fragmentation studies.

### Tree species surveys and habitat classification

We compared the frequencies and the functional diversity of tree reproductive traits in 10 of the forest fragments (range = 3.4–295.7 ha) and 10 ‘forest interior plots’ [*sensu* 41] located in the region's largest remnant (Coimbra forest; here adopted as the control site) using floristic data from previously conducted botanical surveys. Although we are aware that the Coimbra forest does not represent a true ‘continuous forest’, it is the largest remaining Atlantic forest patch in Northeast Brazil [see 19] and is more than twice as large as the largest fragment analyzed by Ranta *et al*. [33] in this same center of endemism. In addition, the Coimbra forest still retains the full complement of ecological groups occurring in more continuous tracts of Atlantic forest, such as large-seeded trees and frugivorous vertebrates [16], [17], [42], [43]. It is therefore representative of the largest tracts of forest remaining in the hotspot, making its core area [*sensu* 41] the best possible control site for assessing persistent and long-term effects of habitat loss and fragmentation.

The tree surveys, upon which we randomly selected our fragments and control plots, were carried out from 2003–2005 by Oliveira *et al.* [19] and Grillo [39] as part of a regional plant survey. Briefly, all trees ≥10 cm DBH were measured, marked, and identified in one 0.1-ha plot per fragment. Plots were located in the geographic center of fragments to standardize procedures and minimize edge effects [44]. Depending on the size of the fragment, plots were 60.5–502.77 m from nearest edge. The ten control plots, also measuring 0.1-ha, were haphazardly located in the interior of Coimbra forest at distances 200–1012.73 m from nearest edge, in locations consisting of old-growth forest with no detectable edge effects (*i.e.* forest interior [*sensu* 41]). Vouchers collected by Oliveira *et al.* [19] and Grillo [39] are deposited in the Herbarium UFP (No. 34.445 to 36.120), and the checklist of the flora of Usina Serra Grande (ca. 650 plant species) is available at www.cepan.org.br and in Pôrto *et al.* [45]. Since 2001, the number of botanical investigations carried out in our study landscape has increased [*e.g.* 15], [19], [34], [38], [39], [45], [46], providing detailed knowledge about the taxonomy and life-history traits of the woody flora.

### Reproductive traits of tree species

Floristic surveys revealed a total of 629 individuals from 77 tree species in the forest fragments (32 families, 58 genera), whereas 878 individuals from 119 species (37 families, 87 genera) were recorded in the control plots. Pooling the data from all sites resulted in 1507 individuals from 156 species (41 families, 105 genera) (see Table S1 in the Supporting Information). For each species we identified the following “reproductive traits”: pollination system, floral biology, sexual system, and reproductive system (Table 1). Classification of species into each category was based on (1) floras and botanical monographs [*e.g.* 47], [48]–[50], including several issues of *Flora Neotropica*; (2) web searches including only published and referenced data; (3) field observations and a survey of specimens from the UFP and IPA Herbaria; and (4) personal knowledge and previously published observations [see 51 for a review]. For each fragment and control plot we then calculated the proportion of tree species and individuals within the 50 categories that comprise the four major classes of reproductive traits (Table 1). Although not all categories could be identified for a few of the species (see *results*), it is unlikely that this biases the qualitative outcome of our analyses because habitats were compared in terms of frequency of species and individuals within categories.

**Table 1:** Tree reproductive traits with their respective categories adopted in this study.

Reproductive traits	Categories*
1. Pollination system^1^	bats; bees; beetles; birds; butterflies; diverse small insects (DSI); flies; moths (excluding hawkmoths); Sphingids (hawkmoths); non-flying mammals; wasps; wind
2. Floral biology	
Size^2^	inconspicuous (≤4 mm); small (>4≤10 mm); medium (>10≤20 mm); large (>20≤30 mm); very large (>30 mm)
Reward^1^	brood or mating places/floral tissues (BMFT); nectar; oil; pollen; nectar/pollen; without resource (other than deceit flowers)
Type^3^	bell/funnel; brush; camera; flag; gullet; inconspicuous (attributed to very small flowers, ≤4 mm); open/dish; tube
Anthesis period^1^	diurnal; nocturnal
3. Sexual system^4^ (morphological expression)	andromonoecious; dioecious; hermaphrodites (distinguishing those heterostylous); heterostylous; monoecious
4. Reproductive system^4^,^5^	agamospermic; self-compatible; self-incompatible; outcrossing (self-incompatible+dioecious species)

1 According to [66], [86], [87];

2 According to [88];

3 Adapted from [66];

4 According to [89];

5 Outcrossing (or obligatory xenogamous) according to [90].

* To analyze data we also grouped some categories into new ones as: 1) generalist pollen vectors *sensu* [65] (including small bees, butterflies, DSI, flies, moths, wasps, and wind); 2) specialist pollen vectors *sensu* [65] (including bats, medium-large bees, beetles, birds, hawkmoths, and non-flying mammals); 3) small+inconspicuous flowers; 4) medium+large+very large flowers; 5) open/dish+inconspicuous flowers ( = flowers with easily accessible resource *sensu* [66]); 6) floral types other than open or inconspicuous ( = flowers with concealed resource *sensu* [66]); 7) bird-+bat-+non-flying mammal-pollinated flowers ( = vertebrate pollination); 8) non-hermaphrodite sexual systems.

### Explanatory variables

Because a number of patch and landscape-scale environmental variables may affect the structure of tree assemblages in tropical forests [8], [52], we also considered the effects of soil type, distance to the nearest forest edge, forest fragment size, the spatial distribution of plots (*i.e.* plot location in the landscape), and the amount of forest cover retained in the surrounding landscape (hereafter forest cover) as independent variables for the frequency of reproductive traits in the tree assemblages. Forest cover is positively correlated with overall connectivity between patches [53] and was quantified as the percentage of forest within a 1-km width buffer set from the border of each fragment. Patch and landscape metrics were quantified using a combination of three Landsat and Spot images acquired in 1989, 1998, and 2003, a set of 160 aerial photos (1∶8,000) taken from commissioned helicopter overflights on April 2003, a soil map by IBGE [36], and a soil map provided by the Usina Serra Grande Agriculture Office. Analyses were conducted using ArcView 3.2 and Erdas Imagine 8.4.

### Functional diversity of reproductive traits

Here we operationally define a functional group as a set of tree species within the same category of reproductive trait, *i.e.* a set of species sharing a life-history trait as previously adopted elsewhere [1]. To calculate the functional diversity of reproductive traits in forest fragment and control plots, we used Shannon's (log base 2) and Simpson's indices [54]. We used both indices to elucidate the contribution of both the richness of categories and the evenness to diversity scores (note that the use of evenness-based indices for estimating functional diversity has been recommended by some authors [55]–[57]). We calculated these indices twice for each of the 20 plots: first, using categories as the equivalent of species, and the number of tree species within each category as the equivalent of individuals; and second using categories as the equivalent of species and the number of individual within each category.

### Statistical analysis

Differences in (1) the average percentage of species and individuals within each category of reproductive trait, and in (2) the average functional diversity of reproductive traits between the control area and fragments were compared with *t* or Mann-Whitney tests [58]. General linear models (GLM) were used to detect any effect of explanatory variables on the frequency of traits in tree assemblages by first examining the effects of habitat type (fragments *vs.* control plots), soil type and distance to the nearest edge considering all 20 plots in the two habitats, and then the effects of forest fragment size and surrounding forest cover considering the 10 fragments (since these patches and landscape metrics had no variance in Coimbra forest). Normality of all response variables were checked using Lilliefors tests; for GLMs the percentage-expressed dependent variables were arcsine transformed as suggested by Sokal & Rohlf [58].

Additionally, to examine the effect of habitat and soil type on species similarity between plots these variables were considered as factors in Analysis of Similarities (ANOSIM) tests [59]. Plots were ordered according to their Bray-Curtis dissimilarities of species composition [54]. Species abundance were square-root transformed and standardized [59] to avoid any bias resulting from very abundant species and differences in the sample size of individuals recorded within each plot. We also performed a Mantel test with Weighted Spearman rank correlations to address the effect of plot geographic location on levels of taxonomic similarity. Straight-line distances between plots were ln-transformed, as suggested by Condit *et al.* [60] and Jones *et al.* [61]. The Mantel test was carried out considering a group of 20 fragments and 75 0.1-ha plots from which information on tree species composition is available [19], [39]. Here we assume that the lack of significant relationships between soil type, plot location and plot floristic similarity discard soil and plot location as variables driving the frequency of tree reproductive traits in the landscape. All analyses were carried out using SYSTAT 6.0 [62], PRIMER v. 5 [63], and PC-ORD 4.36 [64].

## Results

### Reproductive traits of tree species

Fragments and control plots differed significantly in more than 50% of the categories of reproductive traits investigated, but differences were much more notable when evaluating individuals within categories (over 60% of the categories differed) than species (ca. 40%).

**Figure 2:**
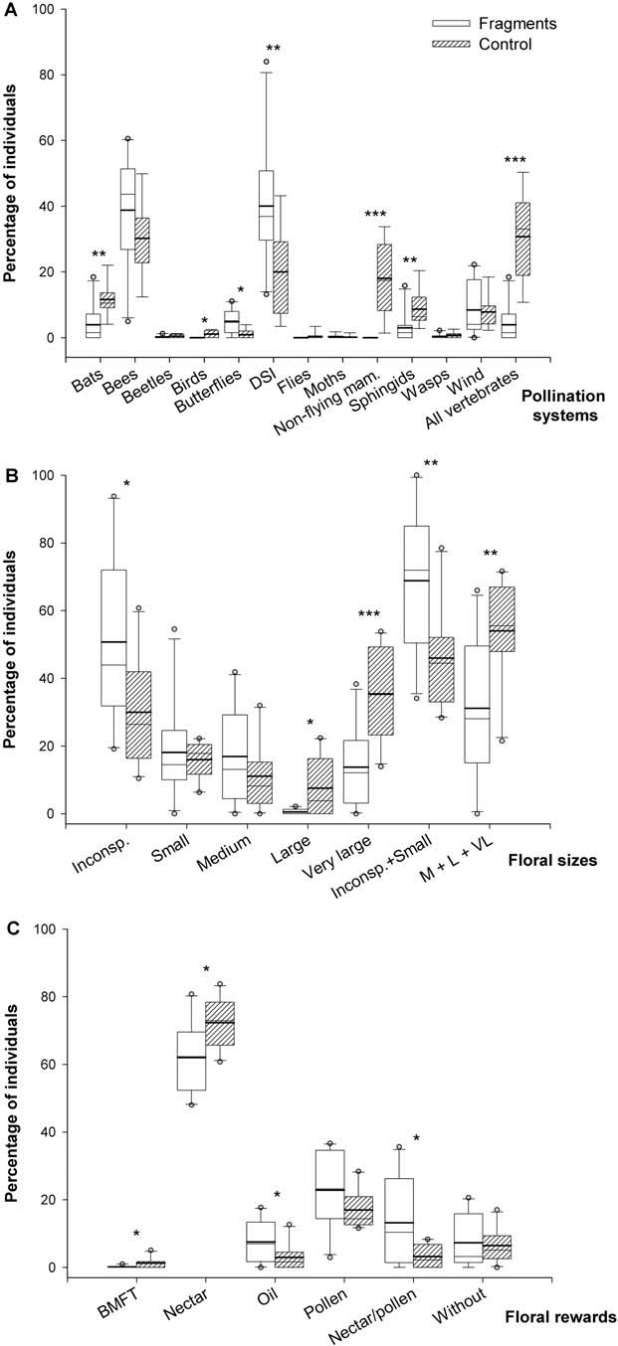
Effect of habitat fragmentation on pollination systems, floral sizes and floral rewards. Percentage of tree individuals within categories of pollination systems (A; N = 137 spp.), floral sizes (B; N = 105 spp.), and floral rewards (C; N = 124 spp.) at 10 fragments and 10 control plots of an Atlantic forest landscape, northeastern Brazil. Frequencies represented by boxes that are significantly different are indicated with asterisks: *P<0.05; **P<0.01; ***P<0.001.

For pollination systems, fragments and control plots markedly differed in 50% of all categories (6 out of 12 categories) (Table 2; Figure 2A). The most conspicuous differences concerning species richness within categories of pollination systems can be summarized in four aspects. First, fragments lacked three categories of pollination systems–pollination by birds, flies and non-flying mammals. Second, scores for hawkmoth- and bat-mediated pollination in fragments were about half of the scores recorded in the control plots. Third, when comparing pollination by vertebrates as whole (birds, bats, and non-flying mammals) fragments had a *ca.* threefold decreased frequency than control plots. Finally, fragments had a 33% increase in the proportion of tree species pollinated by diverse small insects (DSI) in comparison to control plots (Table 2). The proportion of tree individuals within categories of pollination systems showed similar trends (Figure 2A), although for some categories the differences between fragments and control plots were even more dramatic than for species richness (*e.g.* hawkmoth and vertebrate pollination). Fragments and control plots also differed dramatically when pollination systems were pooled into two categories of pollen vectors-generalists and specialists [*sensu* 65]. In summary, fragments had proportionately more tree species pollinated by generalist vectors (66.43±14.08%) than control plots (58.18±7.87%; *t* = 1.616; *d.f*. = 18; *P* = 0.06); the relative abundance individuals pollinated by generalists was also higher in fragments than control plots (71.71±16.5% *vs.* 46.10±15.53, U = 13.0; *P = *0.0052).

**Table 2:** Percentages (mean±SD) of tree species within categories of reproductive traits in forest fragments (N = 10) and control plots (N = 10) in a fragmented landscape of Atlantic forest, northeastern Brazil (data on the reproductive traits for the species are available upon request).

Reproductive traits	Categories	Fragments		Control plots
		%_mean _species±SD		
Pollination systems	Bats	5.79±7.85 a	14.04±3.74 b**	
N = 137 spp.	Bees	37.77±12.72 a	37.95±7.68 a	
	Beetles	0.97±2.15 a	1.20±1.58 a	
	Birds	0.00 a	1.75±1.64 b*	
	Butterflies	5.59±4.00 a	0.83±1.46 b**	
	Diverse small insects	33.62±8.99 a	22.44±8.05 b**	
	Flies	0.00 a	0.29±0.90 a	
	Moths	1.13±2.39 a	0.42±1.32 a	
	Non-flying mammals	0.00 a	3.32±1.00 b***	
	Sphingids	4.76±5.65 a	8.94±3.60 b*	
	Wasps	1.17±2.49 a	2.22±2.68 a	
	Wind	9.22±7.33 a	6.62±3.72 a	
	All Vertebrates	5.79±7.85 a	19.10±4.70 b**	
Floral sizes (mm)	Inconspicuous (≤4)	42.39±19.01 a	37.31±8.59 a	
N = 105 spp.	Small (>4≤10)	21.22±11.23 a	25.31±6.82 a	
	Medium (>10≤20)	15.02±6.50 a	10.48±6.18 a	
	Large (>20≤0)	1.93±3.26 a	5.77±5.57 a	
	Very Large (>30)	19.44±11.51 a	21.12±4.77 a	
	Inconspicuous+Small	63.61±15.66 a	62.63±8.61 a	
	Medium+Large+Very large	36.39±15.66 a	37.37±8.61 a	
Floral rewards	Brood or mating places/floral tissues	1.02±2.28 a	2.88±2.60 a	
N = 124 spp.	Nectar	62.51±8.16 a	65.50±6.51 a	
	Oil	5.68±2.40 a	2.94±3.04 b*	
	Pollen	24.03±8.99 a	24.24±5.16 a	
	Nectar/pollen	7.79±4.90 a	3.90±3.08 b*	
	Without	6.75±5.52 a	4.44±2.38 a	
Floral types	Bell/funnel	3.39±3.74 a	1.72±2.42 a	
N = 111 spp.	Brush	8.36±7.51 a	22.34±6.80 b***	
	Camera	9.29±7.13 a	10.03±4.40 a	
	Flag	3.13±5.17 a	11.75±5.21 b**	
	Gullet	9.40±7.40 a	0.32±1.02 b**	
	Inconspicuous	36.40±19.70 a	24.61±7.56 b*	
	Open/dish	22.97±8.94 a	18.68±5.55 a	
	Tube	7.06±5.96 a	10.55±2.80 a	
	Inconspicuous+Open	59.37±13.44 a	43.29±6.31 b**	
	All non-inconspicuous or open	40.63±13.44 a	56.71±6.31 b**	
Anthesis period	Diurnal	91.83±9.21% a	80.42±6.44 b**	
N = 116 spp.	Nocturnal	8.17±9.21% a	19.58±6.44% b**	
Sexual systems	Andromonoecious	0.91±1.92 a	0.00±0.00 a	
N = 129 spp.	Dioecious	27.95±7.94 a	31.80±5.48 a	
	Hermaphrodite	65.55±10.80 a	60.28±6.34 a	
	Heterostylous	0.45±1.44 a	0.63±1.37 a	
	Monoecious	5.14±5.05 a	7.29±4.15 a	
	All non-hermaphrodite	34.45±10.80 a	39.72±6.34 a	
Reproductive systems	Agamospermic	0.92±2.92 a	2.74±5.79 a	
N = 79 spp.	Self-compatible	15.51±7.52 a	5.86±9.82 b*	
	Self-incompatible (SI)	51.77±9.27 a	63.44±14.95 b*	
	Outcrossing (SI+Dioecious)^1^	83.57±9.50 a	91.39±14.66 b*	

Values in the same row followed by different letters are significantly different (

* *P*<0.05;

** *P*<0.01

*** *P*<0.001)

1 According to [90].

The proportion of species within categories of floral size was similar in fragments and control plots (Table 2). However, fragments had twice as many individuals with inconspicuous flowers than control plots (50.75±25.44% *vs*. 29.99±15.86%; Figure 2B). An opposite trend was observed for large and very large flowers, fragments with more than a 10-fold lower proportion of individuals with large flowers (0.5±0.84%) and almost a three-fold decrease of the very large ones (13.74±11.77%) in comparison to control plots (7.54±8.58% and 35.4±13.54%, respectively). By grouping the five categories of flower size into two [*i.e.* inconspicuous/small (≤10 mm) and medium/very large (>10 mm)] results were similar. Fragments showing a prevalence of individuals with inconspicuous/small flowers (68.85±21.43%) in contrast with control plots (45.98±16.04%), and a significant lower proportion of individuals with medium to very large flowers ones (31.15±21.43%) than control sites (54.02±16.04%) (Figure 2B).

Nectar was the most frequent floral reward observed in tree species of fragment and control sites, however, these habitats differed in two of the other five categories of floral rewards adopted in this study (Table 2). Nectar/pollen-flower species were twice as higher in fragments than in control plots, and fragments had also higher frequency of species with oil-flowers in comparison with control plots (Table 2). Similar patterns were observed with respect to the proportions of individuals within categories of floral rewards in each habitat, but, additionally, fragments faced a slight and statistically significant reduction on the proportion of individuals with BMFT flowers (0.19±0.41%) in contrast with control plots (1.25±1.56%) (Figure 2C).

**Figure 3:**
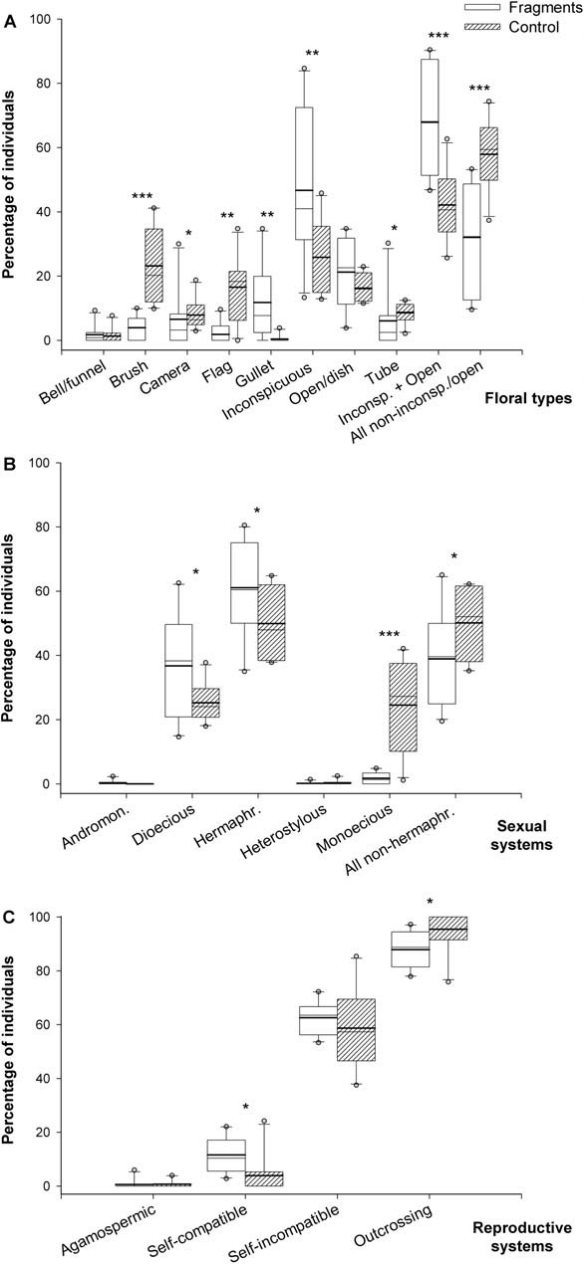
Effect of habitat fragmentation on floral types, sexual systems and reproductive systems. Percentage of tree individuals within categories of floral types (A; N = 111 spp.), sexual system (B; N = 129 spp.), and reproductive system (C; N = 79 spp.) at 10 fragments and 10 control plots of an Atlantic forest landscape, northeastern Brazil. Frequencies represented by boxes that are significantly different are indicated with asterisks: *P<0.05; **P<0.01; ***P<0.001.

As expected, fragments and control plots largely differed in terms of floral types considering the proportion of both species and individuals. Noticeable differences refer to significantly lower scores of species with flag and brush flowers, and higher scores of inconspicuous flowers in fragments in comparison with control plots (Table 2). Similar patterns were detected by analyzing the eight categories of floral types based on reward accessibility: (1) inconspicuous+open/dish flowers (easily accessible resource [*sensu* 66]), and (2) non-inconspicuous/open (concealed resource, at least some degree of hiddenness [*sensu* 66]). Under this approach, fragments showed a prevalence of species with inconspicuous/open type, which was significantly higher than in control sites. In terms of relative abundance of tree species within floral types categories, figures described fragments facing the same patterns observed to species regarding flag, inconspicuous (with even stronger differences), and brush flowers. Additionally, fragments showed lower proportions of individuals bearing camera and tube flowers in contrast with control areas (Figure 3A). Similarly, when observing proportions of individuals within categories of floral types according to reward accessibility, fragments had significant higher frequency of individuals with flowers of the inconspicuous/open type than control plots (Figure 3A), differences being yet more expressive than for species richness. Moreover, fragments revealed to be particularly impoverished in terms of tree species with nocturnal anthesis, showing a frequency more than two times lower (8.17±9.21%) than control plots (19.58±6.44%) (*t = * −3.211; *d.f.* = 18; *P* = 0.002). Difference was even more marked when the relative abundance of tree species with nocturnal anthesis is analyzed (4.93±6.67% in fragments *vs.* 21.18±11.41% in control plots) (*t* = −3.889; *d.f*. = 18; *P* = 0.001).

Both habitats, fragments and control, were dominated by hermaphrodite species and showed similar scores for species within the five categories of sexual systems (Table 2). However, habitats were absolutely contrasting with respect to the frequency of individuals, as fragments were dominated by hermaphrodite individuals (61.05±15.33%), whereas non-hermaphrodite systems were prevalent (50.12±10.66%) among individuals of the control plots. Particularly expressive, as well, was the lower representation of monoecious individuals in the fragments–more than 12-times lower (1.72±1.84%) than control plots (24.51±13.92%) (Figure 3B). Fragments also had a slight but statistically significant decrease in the proportion of self-incompatible and overall obligatory outcrossing species (self-incompatible+dioecious; Table 2). In terms of the relative abundance of tree species within categories of reproductive system, fragments had significant lower scores of outcrossing individuals (87.82±6.84% *vs.* 95.40±8.54 in control plots) and highest frequency of self-compatible ones (11.59±6.56% *vs.* 3.90±8.03 in control sites) (Figure 3C).

### Explanatory variables

GLMs did not reveal any significant influence of soil type on the proportion of traits in tree assemblages. Habitat was consistently the strongest explanatory variable for the proportion of tree species and individuals within categories of reproductive traits, explaining between 19.4% and 69.4% of their variation, influencing 38 categories (Table 3). GLMs also detected 10 categories of reproductive traits that were influenced by log-distance to edge (considering forest fragments and control plots), two categories influenced by log-fragment area, and eight affected by forest cover (considering forest fragments only) (Table 3). These three fragmentation-related variables explained between 20.7% and 68.6% of the variation on reproductive traits in forest fragments and control plots (Table 3). Additionally, ANOSIM revealed no significant correlation between soil type and level of taxonomic similarity between plots (*R* = 0.024; *P* = 0.54), but detected a stronger effect of habitat type (*R* = 0.95; *P* = 0.001). A Mantel test failed to uncover any spatial effects on the taxonomic similarity among plots (*Rho* = 0.155; *P* = 0.9).

**Table 3:** Scores from General Linear Models applied to the proportion of tree species and individuals within categories of reproductive traits (48 categories for species, 48 categories for individuals) in forest fragments (N = 10) and control plots (N = 10) in a fragmented landscape of Atlantic forest, northeastern Brazil.

Habitat/explanatory variables	Traits analyzed	Traits affected	*P* values	R^2^ range
Fragments+control plots				
Habitat	96	38	<0.0001–0.04	19.4–69.4%
Soil	96	0		0
Log-distance to edge	96	10	0.008–0.044	20.7–46.5%
Total		48		
Fragments				
Log-fragment area	96	2	0.014–0.018	52.7–55.4%
Forest cover	96	8	0.003–0.046	39.9–68.6%
Total		10		

### Functional diversity of reproductive traits

When using the number of reproductive categories (see Table 1) and the species richness per category, fragments were significantly less diversified (H′) with respect to pollination systems (−18.4%) and floral types (−12.65%) in comparison with control plots (Table 4). Simpson's values also evidenced fragments with significant lower functional diversity of pollination systems (Table 4). Differences were much more expressive, both biologically and statistically, when using number of categories (as equivalent of species) and number of individuals within categories for calculating diversity indices. In this case, fragments were significantly less diversified (H′ scores) not only in terms of pollination systems (−30.3%) and floral types (−23.6%), but they also presented significant lower functional diversity of floral sizes (−20.8%) in contrast with control plots (Table 4). Simpson's values also evidenced fragments with significant reduced functional diversity of pollination systems (−20.7%) and floral types (−19.62%) (Table 4). Based on Simpson's index, fragments were slightly more diversified than control plots in terms of floral rewards, however, when applying Bonferroni correction, values for floral rewards were not significantly different any more (Table 4).

**Table 4:** Functional diversity (mean±SD) of pollination systems, floral size, floral type and floral reward categories in tree assemblages of forest fragments (N = 10) and control plots (N = 10) in a fragmented landscape of Atlantic forest, northeastern Brazil.

Functional Diversity	Treatments (N = 10 plots/treatment)	Pollination systems (mean±SD)	Floral sizes (mean±SD)	Floral types (mean±SD)	Floral rewards (mean±SD)
Categories and species					
Shannon's (H′)	Fragments	1.965±0.341 a	1.752±0.414 a	2.169±0.429 a	1.386±0.189 a
	Control	2.407±0.213 b***	1.983±0.169 a	2.483±0.168 b**	1.323±0.238 a
Simpson's (1-D)	Fragments	0.732±0.073 a	0.713±0.121 a	0.782±0.126a	0.562±0.069a
	Control	0.781±0.047 b*	0.758±0.053 a	0.843±0.031a	0.521±0.073a
Categories and individuals					
Shannon's H′	Fragments	1.672±0.358 a	1.485±0.567 a	1.810±0.506 a	1.332±0.242 a
	Control	2.398±0.207 b***	1.875±0.161 b*	2.369±0.244 b**	1.167±0.258 a
Simpson's (1-D)	Fragments	0.613±0.130 a	0.566±0.220 a	0.635±0.181 a	0.528±0.100 a
	Control	0.773±0.050 b**	0.695±0.058 a	0.790±0.048 b**	0.437±0.090 b*
Total no. of categories	Fragments	5.4±1.43	4.0±0.94	5.7±1.16	3.9±0.74
	Control	8.0±0.82	4.6±0.52	6.4±0.84	4.1±0.74
Total no. of species	Fragments	18.3±5.81	12.8±4.26	13.4±4.17	17.3±5.48
	Control	32.9±10.54	22.0±7.94	23.3±7.8	28.0±8.10

Diversity was calculated based on categories and species and categories and individuals.

Values in each pair of line of the same column followed by different letters are significantly different (*P*<0.05; *P*<0.01; *P*<0.001); *When applying Bonferroni correction, values followed by one asterisk (*P*<0.05) are not significantly different.

## Discussion

### Patterns and underlying mechanisms

Our findings suggest that habitat fragmentation promotes marked changes in both the presence and relative abundance of the reproductive traits of tree species, resulting in a reduced functional diversity of tree assemblages in forest fragments. Moreover, small forest patches in severely-fragmented landscapes may be strongly impoverished in terms of the number of species and individuals with particular pollination systems (*e.g.* pollination by bats, birds, non-flying mammals, Sphingids) and may be dominated by tree species pollinated by generalists. Finally, strategies that are more dependent on long-distance pollen movement and animal-mediated services, such as self-incompatibility, may be negatively affected. These statements are supported by the fact that the differences we found between fragments and control plots could not be explained by soil type or the relative spatial position of the plots in the landscape. Although the distribution of tropical trees has been found to be influenced by variation in soil types [52], [67], there is no evidence that this also influences the spatial distribution of ecological groups (based on reproductive traits, regeneration strategy, etc.) in *terra firme* forests [8], [68].

An increasing body of evidence has shown that as fragments become older, tree assemblages become drastically altered [12], [69]–[71]. Plant assemblages in small fragments (<10 ha) and forest edges are impoverished (scores of alpha diversity reduced by a half) and biased in taxonomic and ecological terms towards pioneer species. These patch-level findings suggest that fragmented landscapes tend to retain just a small subset of species from the original biota. Despite the recent findings on this topic, our study is one of the first to document a marked shift on the signature of tree assemblages inhabiting a fragmented landscape with respect to the frequency of reproductive-related traits and its functional diversity. Similar results were reported by Chazdon *et al.* [72] for tree assemblages in second-growth, logged, and old-growth forests in Costa Rica. They found lower relative abundance of mammal-pollinated trees in second-growth forests in comparison to old-growth ones, as well as a higher relative abundance of hermaphroditic trees in second-growth forests. In addition, Murcia [27] suggested fragmented forests tended to have an increased frequency of self-compatible hermaphrodites at the expense of other sexual systems. Our findings are consistent with these results, as well as recent ones indicating self-incompatible systems are more negatively affected than self-compatible ones following habitat loss and fragmentation [12], [25], [29].

Two fragmentation-related processes may be the principal mechanisms driving the changes in reproductive traits and functional diversity we observed: 1) the proliferation of pioneer species with a concomitant decline in the abundance of shade-tolerant trees and 2) depressed population sizes of animal pollinators, which over time led to changes in tree abundance in forest fragments. In tropical forests, myriad processes triggered by the creation of forest edges promote a proliferation of short-lived pioneers [8] and the local extirpation of shade-tolerant trees, including canopy and understory species [10], [19], emergent trees [73] and large-seeded trees [15], [34]. In our study site, pioneer species represent over 80% of all tree species and individuals recorded in the fragments, whereas they represent less 50% in core areas [19], [39]. Furthermore, recent surveys in this site have documented an outstanding predominance of pioneer species in seed rain [15] and seedling assemblages [34], [74] which suggests that pioneer dominance may represent a more pervasive, long-term feature of old and severally fragmented landscapes.

Assuming that pioneer plants are *r*-strategists and shade-tolerant (climax) ones are *K-* strategists [21], it is reasonable to expect that these two species groups differ in terms of reproductive traits, sexual systems, and reproductive systems. Some of our findings, such as higher scores of pollination by DSI and flowers with easily accessible resources (inconspicuous+open/dish flowers) in fragments, may simply reflect the dominance of pioneer trees in this habitat as these traits appear to be more frequent among pioneers (65% of the DSI-pollinated species and over 68% of the species with inconspicuous/open/dish flowers are pioneers). On the other hand, a trait such as pollination by bats that was significantly more frequent in control plots (richness and abundance) is also positively associated with a subset of shade-tolerant species–75% of the bat-pollinated species are shade-tolerant (*e.g. Bauhinia, Hymenaea-*Fabaceae; *Manilkara-*Sapotaceae; *Quararibea-*Malvaceae *sensu* APG II [75]). Because the pioneer species recorded in the fragments - including both short- and long-lived pioneers - belong to 16 orders and eight superordinal clades (*sensu* APG II [75]), the patterns documented here cannot be explained by phylogenetic clustering among pioneers. Even pioneer species that were recorded exclusively in forest fragments belong to four families in four orders and three superordinal clades. Unfortunately, because of the large number of categories for each reproductive trait and the low number of tree species within each category, it was not possible to properly test trait-associated differences between pioneer and shade-tolerant tree species.

In tropical forests, 98–99% of the flowering plant species (and 97.5% of the trees) rely on biotic vectors such as insects and vertebrates for successful pollination [76], [77], and it has been broadly assumed that plant-pollinator interactions are largely detrimentally affected by habitat loss and fragmentation [26]–[29], [78]–[81]. Some of the changes we documented in our fragments are therefore expected, particularly the lack or reduced occurrence of some pollination systems [27], [28], [82]. For instance, fragmented habitats may support less pollinators than continuous habitats due to limited resource availability for pollinators (area-related effects on animal populations). In turn, plants can have a depressed reproductive output as consequence of changes in pollinator diversity, composition, or behavior [25], [28], *i.e.*, reproductive impairment driven by pollination limitation [*sensu* 29]. Studies on pollinator diversity carried out in our landscape have documented a decreased diversity of nectarivorous bats [83] and hawkmoths [84] in small fragments. However, empirical evidence to determine which pollination-related traits and plant-pollinator mutualisms are particularly susceptible to habitat disturbance is still scarce [28]. Our results suggest that the reduced number of tree species and individuals pollinated by bats and Sphingids in fragments and the absence of fly-, bird-, and non-flying-mammal-pollinated trees, together with the changes in floral traits and sexual systems, may be a higher order effect promoted by habitat fragmentation.

### Implications of reduced functional diversity

The reduced reproductive functional diversity documented in our study landscape's fragments resulted primarily from the lack or skewed representation of some pollination systems, floral types, and floral size categories in terms of both species and individual (see Table 3). In other words, tree assemblages in this habitat appear to carry a narrower range of floral traits and pollination systems in comparison to patches of forest interior, particularly for pollinators such as mammals and hawkmoths (reduced support capacity). Regardless the underlying mechanism, this narrow range may (1) promote the collapse of pollinator populations; (2) restrict the ecological range of plant and animal groups able to colonize remaining patches of forest or even turn fragments into sink habitats for both plants and their pollinators; and (3) alter the course of natural regeneration or the dynamics of forest fragments toward the establishment of impoverished assemblages in terns of species richness, ecological composition and trophic structure.

Unfortunately, few studies have addressed shifts on the diversity of plant reproductive traits in human-disturbed habitats, especially those traits associated with plant-pollinator interactions [1], [72]. Studies linking these shifts to functional diversity are even more scarce [1], [2], despite the fact that pollination processes influence biodiversity maintenance and ecosystem functioning. Fontaine *et al.* [2], for example, argued that even simple structured plant-pollinator communities may have their persistence threatened due to reduced functional diversity, thereby suggesting that functional diversity of pollination networks is critical to avoid biodiversity loss.

In summary, it is reasonable to propose as a working hypothesis that the persistence of biodiversity and consequently the long-term conservation value of isolated tropical forest fragments may be negatively affected by reduced functional diversity to such an extent yet not anticipated by conservation biologists. Collectively, the proliferation of pioneer species, extirpation of shade-tolerant trees, and reduced functional diversity have the potential to disrupt some trophic interactions [*e.g.* 85]; even landscapes such as ours that were fragmented long ago and are dominated by pioneers may face future biodiversity loss. We believe it would be beneficial for future research to 1) validate and assess the generality of both the patterns and the underlying mechanisms observed here and 2) address more ecosystem level effects driven by reduced functional diversity in fragmented landscapes, such as changes in biodiversity persistence, primary productivity, nutrient cycling, succession, and ecosystem resilience.

## Supporting Information

Table S1 Species studied and their abundance in forest fragments (N = 10) and control plots (N = 10) in a fragmented landscape of Atlantic forest, northeastern Brazil.
(0.55 MB DOC)
