# Supplementary material for: Changes in Tree Reproductive Traits Reduce Functional Diversity in a Fragmented Atlantic Forest Landscape
Source: PLoS One. 2007 Sep 19;2(9):e908. doi: 10.1371/journal.pone.0000908 (PMC1975471; doi:10.1371/journal.pone.0000908)
Supplement: Table S1 — Species studied and their abundance in forest fragments (N = 10) and control plots (N = 10) in a fragmented landscape of Atlantic forest, northeastern Brazil. (0.55 MB DOC) [file pone.0000908.s001.doc]

Table S1. Species studied and their abundance in forest fragments (N= 10) and control plots (N= 10) in a fragmented landscape of Atlantic forest, northeastern Brazil.

| **Family** | **Species** | **Fragment plots** | | | | | | | | | | **Control plots** | | | | | | | | | |
| --- | --- | --- | --- | --- | --- | --- | --- | --- | --- | --- | --- | --- | --- | --- | --- | --- | --- | --- | --- | --- | --- |
|  |  | **1** | **2** | **3** | **4** | **5** | **6** | **7** | **8** | **9** | **10** | **1** | **2** | **3** | **4** | **5** | **6** | **7** | **8** | **9** | **10** |
| Anacardiaceae | *Tapirira guianensis* Aubl. | 21 | 1 | 11 |  | 8 |  | 17 |  | 13 | 3 | 4 |  | 2 |  |  | 1 | 1 |  |  | 2 |
| Anacardiaceae | *Thyrsodium spruceanum* Benth. |  |  | 6 |  |  |  | 9 |  | 1 | 7 | 13 |  | 2 |  |  | 4 |  |  |  | 4 |
| Annonaceae | *Guatteria australis* St. Hil. |  |  |  |  |  |  |  |  | 1 |  |  |  |  |  |  |  |  |  | 1 | 1 |
| Annonaceae | *Xylopia frutescens* Aubl. |  |  |  |  |  |  | 1 |  |  |  |  |  |  |  |  |  |  |  |  |  |
| Annonaceae | *Xylopia ochrantha* Mart. |  |  |  |  |  |  |  |  |  |  |  |  | 1 |  |  |  |  |  |  |  |
| Apocynaceae | *Aspidosperma discolor* A. DC. |  |  |  |  |  |  |  |  |  |  | 1 |  | 3 |  |  | 1 |  |  |  | 1 |
| Apocynaceae | *Aspidosperma spruceanum* Benth. ex Müll. Arg. |  |  |  |  |  |  |  |  |  |  | 1 |  | 1 |  |  | 1 |  |  |  | 1 |
| Apocynaceae | *Himatanthus bracteatus* (A.DC.) Woodson |  |  | 1 |  |  |  | 3 |  | 2 | 7 | 4 |  |  |  |  | 5 |  |  |  |  |
| Apocynaceae | *Tabernaemontana flavicans* Willd. ex Roem. & Schult. |  |  |  | 6 |  |  |  |  |  |  |  |  |  |  |  |  |  |  |  |  |
| Aquifoliaceae | *Ilex* aff. s*apotifolia* Reiss. |  |  |  |  |  |  |  |  |  | 1 |  |  |  |  |  |  |  |  | 2 |  |
| Araliaceae | *Schefflera morototoni* (Aubl.) Maguire, Steyerm. & Frodin |  |  |  |  | 5 |  | 4 |  |  |  | 6 |  |  |  |  | 5 |  |  |  | 2 |
| Arecaceae | *Attalea oleifera* Barb.Rodr. |  |  |  |  |  |  |  |  | 2 | 1 |  |  |  |  |  |  |  |  |  |  |
| Arecaceae | *Elaeis guianensis* Steud. |  | 2 |  |  |  |  |  |  |  |  |  |  |  |  |  |  |  |  |  |  |
| Arecaceae | *Euterpe* sp. |  |  |  |  |  |  |  |  |  |  |  |  |  |  |  |  | 1 |  |  |  |
| Bignoniaceae | *Tabebuia avellanedae* Lorentz ex Griseb. |  |  |  | 1 |  |  |  |  |  |  |  |  |  |  |  |  |  |  |  |  |
| Bignoniaceae | *Tabebuia chrysotricha* (Mart.ex DC.) Standl. |  | 1 |  | 4 |  |  |  |  | 2 |  |  |  |  |  |  |  |  |  |  | 1 |
| Boraginaceae | *Cordia sellowiana* Cham. | 7 | 3 | 1 |  | 5 |  | 1 | 4 |  |  |  |  |  |  |  |  |  |  |  |  |
| Boraginaceae | *Cordia trichotoma* Vell. ex Steud. |  |  |  | 7 |  |  |  |  |  |  |  |  |  |  |  |  |  |  |  |  |
| Burseraceae | *Protium aracouchini* (Aubl.) March. |  |  |  |  |  |  |  |  | 1 |  |  |  |  |  |  | 2 | 1 |  |  |  |
| Burseraceae | *Protium giganteum* Engl. |  |  |  |  |  |  |  |  | 7 |  | 3 |  | 1 |  |  | 1 |  |  | 1 |  |
| Burseraceae | *Protium heptaphyllum* March. |  |  |  |  |  |  |  |  | 2 |  | 1 |  |  |  |  |  |  |  | 5 | 3 |
| Burseraceae | *Tetragastris* sp.1 |  |  |  |  |  |  |  |  |  |  |  |  |  |  |  |  |  | 1 | 1 |  |
| Burseraceae | *Tetragastris* sp.2 |  |  |  |  |  |  | 1 |  |  |  |  |  |  |  |  |  |  |  |  |  |
| Chrysobalanaceae | *Couepia impressa* Prance |  |  |  |  |  |  |  |  |  | 2 |  |  | 1 |  | 2 | 2 |  | 2 |  |  |
| Chrysobalanaceae | *Couepia rufa* Ducke |  |  |  |  |  |  |  |  |  |  | 2 | 2 |  |  | 2 | 1 |  |  |  | 1 |
| Chrysobalanaceae | *Hirtella hebeclada* Moric. |  |  |  |  |  |  |  |  |  |  |  | 1 |  |  |  |  |  |  |  |  |
| Chrysobalanaceae | *Licania belemii* Prance |  |  |  |  |  |  |  |  |  |  |  | 1 |  | 2 | 1 |  | 4 | 3 | 3 |  |
| Chrysobalanaceae | *Licania kunthiana* Hook.f. |  |  |  |  |  |  |  |  |  |  | 2 |  |  |  |  |  |  |  | 3 | 1 |
| Clusiaceae | *Rheedia brasiliensis* Planch. & Triana |  |  |  |  |  |  |  |  |  |  | 1 |  |  |  |  | 1 |  |  | 1 | 1 |
| Clusiaceae | *Rheedia gardneriana* Planch. & Triana |  |  |  |  |  |  |  |  |  |  |  |  |  |  | 3 |  |  |  |  |  |
| Clusiaceae | *Symphonia globulifera* L.f. |  |  |  |  |  |  |  |  |  |  | 3 |  |  |  | 1 | 3 | 2 |  | 1 | 1 |
| Clusiaceae | *Tovomita mangle* G.Mariz |  |  |  |  |  |  |  |  |  |  | 2 | 1 | 7 |  | 2 |  |  | 5 | 1 |  |
| Combretaceae | *Buchenavia capitata* Eichl. |  |  |  |  |  |  |  |  |  |  |  |  |  | 1 |  |  |  |  |  |  |
| Elaeocarpaceae | *Sloanea guianensis* Benth. |  |  |  |  |  |  |  |  |  |  | 2 |  |  |  |  |  |  |  |  | 1 |
| Elaeocarpaceae | *Sloanea obtusifolia* K.Schum. |  |  |  |  |  |  |  |  |  | 1 |  | 1 | 1 | 1 | 1 |  | 1 | 2 |  |  |
| Euphorbiaceae | *Croton floribundus* Spreng. |  |  |  |  |  |  |  |  | 1 | 2 |  |  |  |  |  |  | 6 | 1 |  |  |
| Euphorbiaceae | *Hyeronima alchorneoides* Allem. |  |  |  |  |  |  |  |  |  |  | 1 |  |  |  |  | 3 |  |  |  | 1 |
| Euphorbiaceae | *Mabea occidentalis* Benth. |  |  |  |  |  |  |  |  |  |  | 1 | 11 | 11 | 32 | 5 | 8 | 22 | 23 | 25 | 18 |
| Euphorbiaceae | *Senefeldera multiflora* Mart. |  |  |  |  |  |  |  |  |  | 1 |  |  |  |  |  |  |  | 1 |  | 5 |
| Fabaceae | *Abarema cochliacarpos* (Gomes) Barneby & J.W.Grimes |  |  | 1 |  |  |  |  |  |  |  |  |  |  |  |  |  |  |  |  |  |
| Fabaceae | *Andira* aff. p*aniculata* Benth. |  |  |  |  |  |  |  |  |  |  | 1 |  | 1 |  |  | 2 |  |  |  | 2 |
| Fabaceae | *Balizia pedicellaris* (DC.) Barneby & J.W.Grimes |  |  |  |  |  |  |  |  |  |  | 2 |  |  |  |  | 3 |  |  | 1 |  |
| Fabaceae | *Bauhinia forficata* Link |  |  |  |  |  |  |  |  |  |  |  |  |  |  |  |  |  |  |  | 2 |
| Fabaceae | *Bowdichia virgilioides* H.B. & K. |  |  | 5 | 1 | 15 | 1 |  | 2 | 5 | 3 |  |  |  |  |  |  |  |  |  |  |
| Fabaceae | *Chamaecrista ensiformis* (Vellozo) H.S.Irwin & Barneby |  |  |  |  |  |  |  |  |  |  | 2 |  | 1 | 5 | 2 | 9 | 7 | 5 | 2 |  |
| Fabaceae | *Copaifera langsdorffii* Desf. |  |  |  |  |  |  |  |  |  |  |  |  |  |  |  | 1 |  |  | 1 |  |
| Fabaceae | *Dialium guianense* Steud. |  |  |  |  |  |  |  | 5 |  |  |  |  |  |  | 1 | 1 | 3 | 1 |  |  |
| Fabaceae | *Diplotropis purpurea* (Rich.) Amshoff |  |  |  |  |  |  |  |  |  |  | 1 |  | 1 |  |  | 5 |  |  |  | 1 |
| Fabaceae | *Hymenaea courbaril* L. |  |  |  |  |  |  |  |  |  |  |  |  |  |  |  |  |  | 1 | 1 |  |
| Fabaceae | *Inga blanchetiana* Benth. |  |  | 1 |  |  |  |  |  |  |  | 1 | 1 |  |  |  | 1 |  |  |  | 1 |
| Fabaceae | *Inga dysantha* Benth. |  |  |  |  |  |  |  |  |  |  | 1 |  |  |  |  |  | 2 |  |  |  |
| Fabaceae | *Inga edulis* Mart. |  |  |  |  |  |  |  |  |  |  |  | 9 | 1 | 14 |  |  | 7 | 2 | 6 | 2 |
| Fabaceae | *Inga striata* Benth. |  |  |  |  |  |  |  |  |  |  |  | 1 |  |  |  |  |  |  |  |  |
| Fabaceae | *Inga* cf. *subnuda* Salzm. ex Benth. |  | 1 |  |  |  | 1 |  |  |  |  |  |  |  |  |  |  |  |  |  |  |
| Fabaceae | *Inga thibaudiana* DC. |  |  | 2 |  |  |  | 1 |  |  |  |  |  |  |  |  |  |  |  |  |  |
| Fabaceae | *Inga* sp.1 |  |  |  |  |  |  |  |  | 3 |  |  |  |  |  |  |  |  |  |  |  |
| Fabaceae | *Inga* sp.2 |  |  |  |  |  |  |  |  |  |  |  |  |  |  |  |  | 1 |  |  |  |
| Fabaceae | *Machaerium hirtum* (Vell.) Stellfeld |  | 11 | 2 | 3 | 1 | 3 |  |  |  |  |  |  |  |  |  |  |  |  |  |  |
| Fabaceae | *Parkia pendula* Benth. ex Walp*.* |  |  |  |  |  |  |  |  |  |  |  |  | 1 |  |  |  |  |  |  |  |
| Fabaceae | *Plathymenia foliolosa* Benth. |  |  |  |  |  |  |  |  | 1 |  |  | 1 |  |  |  |  |  |  |  |  |
| Fabaceae | *Pterocarpus violaceus* Vog. |  |  |  |  |  |  |  |  |  |  |  |  | 2 | 2 |  |  | 3 | 3 | 1 |  |
| Fabaceae | *Samanea tubulosa* (Benth.) Barneby & J.W.Grimes |  | 3 |  |  |  | 1 |  |  |  |  |  |  |  |  |  |  |  |  |  |  |
| Fabaceae | *Sclerolobium densiflorum* Benth. |  |  |  |  |  |  |  |  |  |  | 2 |  |  |  |  |  |  |  |  |  |
| Fabaceae | *Stryphnodendron pulcherrimum* Hochr. |  |  | 3 |  |  |  | 16 |  |  | 3 |  |  | 1 |  |  | 1 |  |  |  |  |
| Fabaceae | *Swartzia macrostachya* Benth. | 5 |  |  |  |  | 1 |  | 3 |  |  |  |  |  |  |  |  |  |  |  |  |
| Fabaceae | *Zollernia paraensis* Huber |  |  |  |  |  |  |  |  |  |  |  |  |  |  | 1 |  |  |  |  | 1 |
| Lauraceae | *Cryptocarya* sp. |  |  |  |  |  |  |  |  |  |  |  |  | 1 |  |  |  |  | 1 |  |  |
| Lauraceae | *Nectandra* sp. |  |  |  |  |  |  |  |  |  |  | 2 |  |  |  |  | 3 | 1 | 1 | 1 | 2 |
| Lauraceae | *Ocotea bracteosa* Mez |  |  |  |  |  |  |  |  |  |  | 10 |  |  |  |  |  |  |  |  |  |
| Lauraceae | *Ocotea glomerata* Mez |  |  | 9 | 1 | 2 |  | 19 |  |  |  | 1 |  |  |  |  |  |  |  |  |  |
| Lauraceae | *Ocotea opifera* Mart. |  |  |  |  |  |  |  |  |  |  | 3 |  |  |  |  | 1 |  |  |  |  |
| Lauraceae | *Ocotea* sp.1 |  |  |  |  |  |  |  |  |  |  | 2 |  |  |  |  |  |  |  |  |  |
| Lauraceae | *Ocotea* sp.2 |  |  |  |  |  |  |  |  |  |  | 1 |  |  |  |  | 1 |  |  |  |  |
| Lauraceae | *Ocotea* sp.3 |  |  |  |  |  |  |  |  |  |  |  |  |  |  |  |  |  |  | 3 | 1 |
| Lecythidaceae | *Eschweilera ovata* Mart ex Miers |  |  | 1 |  | 1 |  |  |  | 13 | 13 | 6 |  | 3 |  | 4 | 3 |  | 1 | 6 |  |
| Lecythidaceae | *Eschweilera* sp. |  |  |  |  |  |  |  |  |  |  |  |  |  |  |  |  |  |  |  | 2 |
| Lecythidaceae | *Lecythis* cf. *lanceolata* Poir. |  |  |  |  |  |  |  |  |  |  |  |  |  |  |  | 1 |  |  |  | 1 |
| Lecythidaceae | *Lecythis lurida* (Miers) S.A.Mori |  |  | 1 | 1 | 1 |  |  | 2 | 1 | 4 |  | 1 |  |  | 1 |  |  |  | 2 | 1 |
| Lecythidaceae | *Lecythis pisonis* Cambess. |  |  |  |  |  |  |  | 1 |  | 1 |  |  |  | 1 |  |  | 2 |  |  |  |
| Malpighiaceae | *Byrsonima crispa* A. Juss. |  |  |  |  |  |  |  |  |  |  |  |  | 1 |  |  |  |  |  |  |  |
| Malpighiaceae | *Byrsonima sericea* DC. |  | 2 | 10 | 1 | 3 | 3 | 1 | 6 | 16 | 8 |  |  |  |  |  |  |  |  |  |  |
| Malpighiaceae | *Byrsonima* cf. *stipulacea* A. Juss. |  |  |  |  |  |  |  |  |  | 6 |  |  | 2 |  |  |  |  |  |  |  |
| Malvaceae | *Apeiba tibourbou* Aubl. |  | 3 |  |  | 1 |  |  |  | 1 |  |  |  |  |  |  |  |  |  |  |  |
| Malvaceae | *Eriotheca crenulaticalyx* A.Robyns |  |  |  |  |  |  |  |  |  | 1 |  |  |  |  |  | 2 |  |  |  | 4 |
| Malvaceae | *Eriotheca gracilipes* (K.Schum.) A.Robyns |  |  | 3 |  |  |  |  |  |  |  |  |  |  |  | 1 |  |  |  |  |  |
| Malvaceae | *Guazuma ulmifolia* Wall. |  | 6 |  |  | 2 | 5 |  |  |  |  |  |  | 1 |  |  |  |  |  |  |  |
| Malvaceae | *Luehea speciosa* Willd. |  |  |  |  |  | 5 |  |  |  |  |  |  |  |  |  |  |  |  |  |  |
| Malvaceae | *Quararibea turbinata* Poir. |  |  |  |  |  |  |  |  |  |  |  |  |  |  |  |  | 1 |  |  |  |
| Melastomataceae | *Miconia calvescens* DC. |  |  |  |  |  |  |  |  |  |  | 3 |  |  |  |  | 4 |  |  |  |  |
| Melastomataceae | *Miconia hypoleuca* Triana |  |  |  |  |  |  |  |  |  | 1 |  |  |  |  | 2 |  |  |  |  |  |
| Melastomataceae | *Miconia prasina* DC. |  |  |  |  |  |  |  |  | 1 |  |  |  |  |  |  |  |  |  |  |  |
| Melastomataceae | *Miconia* sp. |  |  | 1 |  |  |  |  |  |  |  |  |  |  |  |  |  |  |  |  |  |
| Meliaceae | *Cabralea canjerana* (Vell) Mart. |  |  |  |  |  |  |  |  |  |  |  | 1 |  |  |  |  |  | 1 |  |  |
| Meliaceae | *Cedrela odorata* Vell. |  |  |  |  |  |  |  |  |  |  |  | 1 |  |  |  |  |  |  |  |  |
| Meliaceae | *Guarea guidonia* (L.) Sleumer |  |  |  |  | 1 |  |  |  |  |  |  |  |  | 4 |  |  | 4 | 3 | 1 |  |
| Meliaceae | *Trichilia lepidota* Mart. |  |  |  |  |  |  |  |  | 3 |  |  |  | 1 |  | 1 | 1 |  |  |  |  |
| Moraceae | *Brosimum guianense* Huber ex Ducke | 1 |  |  |  |  |  | 1 | 1 |  |  |  |  |  |  |  | 2 |  |  |  |  |
| Moraceae | *Brosimum paraense* Huber |  |  |  |  |  |  |  |  |  |  | 3 |  | 1 |  |  | 2 |  |  |  |  |
| Moraceae | *Brosimum rubescens* Taub. |  |  |  |  |  |  |  | 8 |  |  |  |  |  |  |  |  |  |  |  |  |
| Moraceae | *Clarisia racemosa* Ruiz & Pav. |  |  |  |  |  |  |  |  |  |  |  |  | 1 |  |  |  |  | 1 |  | 1 |
| Moraceae | *Ficus gomelleira* Hort.Monac. ex Kunth & Bouche |  |  |  |  |  |  |  |  |  |  |  |  |  |  |  |  |  | 1 |  | 1 |
| Moraceae | *Ficus guaranitica* Chod. |  |  |  |  |  |  |  |  |  |  |  |  |  | 1 |  |  | 1 |  |  |  |
| Moraceae | *Helicostylis tomentosa* (Poepp. & Endl.) Macbride | 11 |  |  |  |  |  |  |  |  |  | 3 | 8 | 6 | 2 | 5 | 10 |  | 2 | 2 | 4 |
| Moraceae | *Sorocea hilarii* Gaudich. |  |  |  |  |  | 2 |  | 2 |  |  |  |  |  |  | 3 |  |  |  |  |  |
| Myristicaceae | *Virola gardneri* Warb. |  |  |  |  |  |  |  |  |  |  | 2 | 1 | 5 | 2 | 4 | 7 | 2 | 3 | 9 | 6 |
| Myrsinaceae | *Myrsine guianensis* (Aubl.) Kuntze |  |  | 2 |  |  |  |  |  | 7 | 1 |  |  |  |  |  |  |  |  |  |  |
| Myrtaceae | *Myrcia fallax* DC. |  |  |  |  |  |  |  |  |  |  | 2 |  | 1 | 2 | 1 |  |  |  |  | 1 |
| Myrtaceae | *Myrcia sylvatica* DC. |  |  |  |  | 2 |  |  |  |  |  |  |  |  |  |  |  |  |  |  |  |
| Myrtaceae | *Psidium* sp. |  |  |  |  |  |  |  |  | 1 |  |  |  |  |  |  |  |  |  |  | 1 |
| Nyctaginaceae | *Guapira opposita* (Vellozo) Reitz |  |  | 5 | 6 | 3 |  |  | 5 |  | 7 |  |  | 1 | 1 |  |  | 2 |  |  | 2 |
| Nyctaginaceae | *Pisonia laxa* Netto |  | 1 |  |  |  |  |  |  |  |  |  |  |  |  |  |  |  |  |  |  |
| Nyctaginaceae | *Pisonia* sp. | 2 |  |  |  |  |  |  |  |  |  |  |  |  |  |  |  |  |  |  |  |
| Ochnaceae | *Ouratea castaneaefolia* Engl. |  |  |  |  |  |  |  |  |  |  | 3 |  |  |  |  |  |  |  |  |  |
| Polygonaceae | *Coccoloba mollis* Casar. |  | 4 |  | 1 |  |  |  |  |  |  |  |  |  |  |  |  |  |  |  |  |
| Polygonaceae | *Coccoloba* cf. *ochreolata* Wedd. |  |  |  |  |  |  |  |  |  |  |  |  |  | 1 |  |  |  |  |  |  |
| Proteaceae | *Roupala* cf. *rhombifolia* Mart. ex Meisn. |  |  |  |  |  |  |  |  |  |  |  |  |  |  |  |  |  |  |  | 1 |
| Quiinaceae | *Quiina paraensis* Pires & Froes |  |  |  |  |  |  |  |  |  |  |  | 1 |  |  |  |  |  |  | 1 | 1 |
| Rhamnaceae | *Ziziphus joazeiro* Mart. |  | 1 |  | 1 |  |  |  |  |  |  |  |  |  |  |  |  |  |  |  |  |
| Rubiaceae | *Alseis floribunda* Schott |  |  |  | 1 |  | 1 |  | 1 | 2 | 5 |  |  |  |  |  |  |  |  |  |  |
| Rubiaceae | *Amaioua* sp. |  |  |  |  |  |  |  |  |  |  |  |  | 2 |  |  |  |  | 1 |  |  |
| Rubiaceae | *Faramea* sp. |  |  |  |  |  |  |  |  |  |  |  | 1 |  |  |  |  |  |  |  |  |
| Rubiaceae | *Psychotria carthagenensis* Jacq. |  |  |  |  |  |  |  |  |  |  |  |  |  |  |  |  |  |  | 2 | 1 |
| Rubiaceae | *Psychotria sessilis* Vell. |  |  |  |  |  |  |  |  |  | 1 |  |  |  |  |  |  |  |  |  |  |
| Rutaceae | *Conchocarpus heterophyllus* (A.St.-Hil.) J.A.Kallunki & J.R.Pirani |  |  |  |  |  |  |  |  |  |  |  |  |  |  |  |  |  |  |  | 3 |
| Rutaceae | *Hortia arborea* Engl. |  |  |  |  |  |  |  |  |  |  | 3 |  |  |  |  |  |  |  |  |  |
| Rutaceae | *Zanthoxylum rhoifolium* Lam. |  |  |  |  |  |  |  |  | 1 |  |  |  |  |  |  |  |  |  |  |  |
| Salicaceae | *Banara guianensis* Aubl. . |  |  |  |  |  |  |  |  |  |  |  |  | 1 |  | 1 |  |  | 1 | 1 |  |
| Salicaceae | *Casearia javitensis* H.B. & K. |  |  |  |  |  |  |  |  |  |  | 1 |  | 3 |  |  |  |  |  |  |  |
| Sapindaceae | *Allophylus edulis* Niederl. |  |  |  |  |  |  |  |  |  |  |  |  | 3 |  |  |  |  |  |  |  |
| Sapindaceae | *Cupania oblongifolia* Mart. |  |  |  |  |  |  |  |  | 1 |  |  |  |  |  |  |  |  |  |  |  |
| Sapindaceae | *Cupania racemosa* Radlk. |  | 3 |  | 8 |  |  |  | 21 | 2 |  | 1 |  |  |  |  |  |  |  |  |  |
| Sapindaceae | *Cupania revoluta* Radlk. | 4 | 6 |  |  |  |  |  | 6 |  |  |  |  |  |  |  |  |  |  |  |  |
| Sapindaceae | *Cupania* sp. | 1 |  |  |  |  |  |  | 2 |  |  |  |  |  |  |  |  |  |  |  |  |
| Sapindaceae | *Dilodendron bipinnatum* Radlk. |  |  |  |  |  |  |  |  |  |  |  |  |  | 1 |  | 1 | 1 |  |  | 1 |
| Sapindaceae | *Talisia elephantipes* Sandwith |  |  |  |  |  |  |  |  |  |  |  |  |  |  |  |  |  |  |  | 1 |
| Sapotaceae | *Chrysophyllum splendens* Spreng. |  |  |  |  |  |  |  |  |  |  | 1 |  |  |  |  | 1 |  |  |  | 11 |
| Sapotaceae | *Manilkara rufula* (Miq.) H.J.Lam |  |  |  |  |  |  |  |  |  |  |  |  |  |  |  | 1 |  |  |  |  |
| Sapotaceae | *Manilkara salzmanii* DC. |  |  |  |  |  |  |  |  |  |  | 2 | 1 |  | 2 | 1 | 3 | 1 |  | 1 | 1 |
| Sapotaceae | *Manilkara* sp. |  |  |  |  |  |  |  |  |  |  |  | 1 |  |  |  |  |  | 4 |  |  |
| Sapotaceae | *Micropholis compta* Pierre |  |  | 2 |  |  |  |  |  |  |  |  |  | 1 |  |  |  |  | 1 |  |  |
| Sapotaceae | *Pouteria bangii* (Rusby) T.D.Penn. |  |  |  |  |  |  |  |  |  |  |  |  |  |  | 7 |  |  |  |  |  |
| Sapotaceae | *Pouteria* aff. *grandiflora* (A.DC.) Baehni |  |  |  |  |  |  |  |  |  | 1 | 2 |  |  |  |  |  |  |  |  |  |
| Sapotaceae | *Pouteria scytalophora* Eyma |  |  |  |  | 1 |  |  |  |  |  | 9 |  | 2 |  |  |  |  |  |  |  |
| Sapotaceae | *Pouteria* sp.1 |  |  |  |  |  |  |  |  |  |  |  |  |  |  |  |  |  |  | 2 |  |
| Sapotaceae | *Pouteria* sp.2 |  |  |  |  |  |  |  |  |  |  |  |  |  |  |  |  |  |  | 2 |  |
| Sapotaceae | *Pouteria* sp.3 |  |  |  |  |  |  |  |  |  |  |  |  |  |  |  |  |  |  | 1 |  |
| Sapotaceae | *Pouteria* sp.4 |  |  |  | 1 |  |  |  |  |  |  |  |  |  | 6 |  | 3 | 2 |  |  |  |
| Sapotaceae | *Pradosia lactescens* Radlk. |  |  |  |  |  |  |  |  |  |  |  |  |  |  | 1 |  |  | 3 | 1 |  |
| Simaroubaceae | *Simarouba amara* Aubl. |  |  |  |  |  |  | 1 |  |  | 1 | 1 |  |  |  | 1 | 2 |  |  |  | 3 |
| Siparunaceae | *Siparuna guianensis* Aubl. |  |  |  |  |  |  |  |  |  |  |  |  | 3 |  |  |  |  |  |  |  |
| Urticaceae | *Cecropia hololeuca* Miq. |  |  |  |  |  |  |  |  |  |  |  | 4 |  | 3 |  | 1 | 3 | 1 |  |  |
| Urticaceae | *Cecropia pachystachya* Trécul | 2 |  |  | 8 |  | 7 | 2 |  |  |  |  |  |  |  |  |  |  |  |  |  |
| Urticaceae | *Pourouma guianensis* Aubl. |  |  |  |  |  |  |  |  |  |  | 2 | 1 |  | 3 |  | 2 |  |  |  |  |
| Verbenaceae | *Aegiphila sellowiana* Cham. | 2 | 1 |  | 2 |  | 4 |  |  |  |  |  |  |  |  |  |  |  |  |  |  |
| Verbenaceae | *Citharexylum myrianthum* Cham. |  | 1 |  |  | 1 |  |  |  |  |  |  |  |  |  |  |  |  |  |  |  |
| Vochysiaceae | *Vochisia oblongifolia* Warm. |  |  |  |  |  |  |  |  |  |  | 1 |  | 6 |  | 13 | 3 |  |  |  | 6 |
